# Dr. Arthur John Lawrence

**Published:** 1908-05

**Authors:** 


DR. ARTHUR JOHN LAWRENCE, of Los Olivos, Cal., a graduate of the University of Buffalo, medical department, 1875, was found burned to death in his cottage at Camp Meeker's, Cal., aged about 60 years.

